# Molecular and phylogenetic analysis of herpesviruses in endangered free-ranging cervids of Chile: ovine gammaherpesvirus-2—A novel threat to wild and domestic animal health in Chilean Patagonia

**DOI:** 10.3389/fvets.2023.1321172

**Published:** 2024-02-01

**Authors:** Ezequiel Hidalgo-Hermoso, Javier Cabello, Rodrigo Lopez, Vicente Vergara-Wilson, Frank Vera, Carola Valencia, Carlos Sanchez, Sebastian Celis, Alejandra Silva, Aintzane Cariñanos, Ismael Barria, Rocio Vicencio, Sebastián Muñoz-Leal, Paula Aravena, Rocio Lagos, Juan Toro-Letelier, Sebastián Verasay-Caviedes, Antonio Garnham, Irene Peña, Fernando Sánchez, Dario Moreira-Arce, Pablo M. Vergara, Raul Alegria-Moran, Galaxia Cortés-Hinojosa

**Affiliations:** ^1^Fundacion Buin Zoo, Buin, Chile; ^2^Centro de Conservación de la Biodiversidad Chiloé-Silvestre, Ancud, Chile; ^3^Aumen ONG, Coyhaique, Chile; ^4^Departamento de Veterinaria, Parque Zoológico Buin Zoo, Buin, Chile; ^5^School of Veterinary Medicine, Facultad de Ciencias de la Naturaleza, Universidad San Sebastian, Puerto Montt, Chile; ^6^Veterinary Medical Center, Oregon Zoo, Portland, OR, United States; ^7^Departamento de Areas Silvestres Protegidas, Corporacion Nacional Forestal, Punta Arenas, Chile; ^8^Departamento de Ciencia Animal, Facultad de Ciencias Veterinarias, Universidad de Concepción, Chillán, Chile; ^9^Laboratorio Clínico, Hospital Veterinario SOS Buin Zoo, Buin, Chile; ^10^Facultad de Cs Veterinarias y Pecuarias, Univeridad de Chile, Santiago, Chile; ^11^Escuela de Medicina Veterinaria, Universidad Mayor, Santiago, Chile; ^12^Facultad de Ciencias de la Vida, Universidad Andrés Bello, Santiago, Chile; ^13^Escuela de Medicina Veterinaria, Facultad de Agronomía e Ingeniería Forestal, Facultad de Ciencias Biológicas, Facultad de Medicina, Pontificia Universidad Católica de Chile, Santiago, Chile; ^14^Departamento de Gestión Agraria, Universidad de Santiago de Chile (USACH), Santiago, Chile; ^15^Institute of Ecology and Biodiversity (IEB), Santiago, Chile; ^16^Escuela de Medicina Veterinaria, Sede Santiago, Facultad de Recursos Naturales y Medicina Veterinaria, Universidad Santo Tomás, Santiago, Chile

**Keywords:** gammaherpesvirus, malignant catarrhal fever, endangered species, pudu, huemul

## Abstract

**Introduction:**

Herpesvirus infections have been highlighted as emerging diseases affecting wildlife health and the conservation of several taxa. Malignant catarrhal fever (MCF) and infectious keratoconjunctivitis (IKC) are two viruses that infect wild ruminants. Nevertheless, epidemiological data on herpesviruses in South American wild ruminants are limited. An outbreak of caprine gammaherpesvirus-2 (CpHV-2) that recently was suspected as the cause of MCF in southern pudus (*Pudu puda*) prompted the need to conduct molecular screenings in Chilean cervids to understand the epidemiology of herpesviruses. The aim of this study was to determine the occurrence and genetic diversity of herpesviruses in free-ranging cervids from Chile.

**Methods:**

Herpesvirus infection was assessed in antemortem blood samples (*n* = 86) from pudus (*n* = 81) and huemuls (*Hippocamelus bisulcus*) (*n* = 5), as well as postmortem samples of spleen (*n* = 24) and lung (*n* = 3) from pudus, using a nested pan-herpesvirus PCR assay.

**Results:**

Combining all suitable sample types, DNA of pudu gammaherpesvirus-1 was detected in five pudues and five huemuls, with an overall prevalence of 9.90% (*n* = 10/101; 95% CI = 5.11–17.87%). One pudu tested positive for ovine gammaherpesvirus-2 (*n* = 1/96; 1.04%; 95% CI = 0.05–6.49%), and one pudu tested positive for a *Macavirus* sequence with 98.63 similarity to ovine gammaherpesvirus-2 (*n* = 96; 1.04%; 95% CI = 0.05–6.49%).

**Discussion:**

To the best of our knowledge, this is the first report of a herpesvirus in huemul and of ovine gammaherpesvirus-2 in Chile. Our results also confirm the active circulation of herpesvirus in free-ranging cervids in Chilean Patagonia, and as such, MCF should be considered as a possible cause of disease in free-ranging Chilean pudus and livestock species. Further research is necessary to develop a plan of systematic monitoring (serological and pathological screening) of herpesviruses in Chilean wild and domestic ruminants to understand their diversity and impact on animal health and conservation.

## 1 Introduction

Herpesviruses are a large group of linear, non-segmented, double-stranded, enveloped DNA viruses that infect many vertebrate and invertebrate species (1). The Herpesviridae family includes three subfamilies, Alphaherpesvirinae, Betaherpesvirinae, and Gammaherpesvirinae, all of which can establish latency in different organs (1, 2). Herpesvirus infections have been cataloged as emerging diseases in wild mammals. For example, hemorrhagic diseases in elephants are caused by elephant endotheliotropic herpesvirus (3, 4), and malignant catarrhal fever (MCF) is caused by several macaviruses in ruminant species (5–8). All these agents can cause devastating illness at the individual or population level and represent a threat to animal health and conservation. In cervids, different species of the Alpha-, Beta-, and Gammaherpesvirinae subfamilies have been described as potential agents of various diseases, including cervid herpesvirus-2 (CvHV-2), which causes infectious keratoconjunctivitis (9), alcelaphine gammaherpesvirus-1 (AlHV-1), alcelaphine gammaherpesvirus-2 (AlHV-2), ovine gammaherpesvirus-2 (OvHV-2), caprine gammaherpesvirus-2 (CpHV-2), hippotragine HV-1 (HiHV-1), ibex-MCFV, and caprine gammaherpesvirus-3, which cause MCF (10–16), and bovine herpesvirus-1, which causes infectious bovine rhinotracheitis (17). Although herpesviruses have been widely investigated, little is known about herpesvirus prevalence and host specificity in cervid species in wild environments. Indeed, to date, most of the reports come from populations under human care (18–21). In Chile, caprine gammaherpesvirus-2 was recently reported in southern pudus (*Pudu puda*) from a zoological collection (or zoo), possibly associated with an MCF outbreak (22). Additionally, a new pudu gamma-herpesvirus-1 was detected in a rescued pudu at Chiloé Island (22). However, there have been no reports of gammaherpesvirus infection or cases of MCF in livestock in Chile to date.

There are three species of cervids native to Chile: the taruka (*Hippocamelus antisensis*) restricted to the north of the country; the huemul (*Hippocamelus bisulcus*) distributed in Patagonia and central Chile; and the southern pudu [with the largest populations throughout temperate coastal and Andean forests (36–49°S)] (23, 24). In Chile, the pudu is classified as vulnerable, and the huemul is classified as endangered (25). The main threats to the conservation of both species include fragmentation of their habitats, traumatic events such as road kills by cars, attacks by dogs, and emerging diseases potentially transmitted via livestock (22, 24, 26–32). The present study aimed to investigate the presence of herpesvirus DNA and genetically characterize it in the blood, spleen, and lung samples from free-ranging southern pudus and huemuls in Chile.

## 2 Materials and methods

### 2.1 Animal sampling

#### 2.1.1 Pudu

Between July 2017 and June 2022, 96 healthy or ill pudus from three wildlife rescue centers, two in the Los Lagos region (USS: Universidad San Sebastian, Ch S: Chiloe Silvestre) and one in the Ñuble region (UC: Universidad de Concepcion), were prospected. Blood or samples of organs were collected on the day of admission or 24 h later during necropsy in the case of animals that perished after being admitted. A physical examination of the animals was performed before taking blood samples. Using a standard vacutainer system (Vacutainer, Beckon, Dickson, and Company, Franklin Lakes, New Jersey, USA), 2–4 mL of blood was collected from the cephalic or saphenous vein and stored at −20°C until processing. This procedure was performed after sedating the animals with 0.04 mg/kg of dexmedetomidine (Dexdomitor, Zoetis, Santiago, Chile), and subsequently, the sedation was reversed with 0.4 mg/kg of atipamezole (Antised, Zoetis), both of which were administered intramuscularly (26). The spleen and lung samples were collected using fine-needle aspirates. None of the animals were euthanized; instead all of them died because of different causes, including trauma, such as being hit by a car, attack by dogs, or presumptive infectious diseases. All samples were stored at −20°C until DNA extraction. According to the guidelines of the authors' institutions, a formal approval by the ethical committee was not required.

#### 2.1.2 Huemul

Blood samples were taken from healthy adult free-ranging huemuls captured in April–May 2019 in Torres del Paine National Park (468010 580 0S, 718580 370 0W), Chilean Patagonia. Their captures were carried out under the supervision of wildlife veterinarians, biologists, and park rangers and authorized by permits 2107/2019 and 256/2019 of the Servicio Agricola y Ganadero and National Forest Corporation, respectively. The animals were anesthetized with a combination of medetomidine (0.09 mg/kg) and ketamine (2 mg/kg) administered intramuscularly (33). Blood was collected from the cephalic vein, placed in EDTA-coated tubes, and kept at −20°C until analysis. All animals were released in the same place immediately after recovery from anesthesia.

### 2.2 Molecular characterization

DNA extractions were carried out using a Quick-DNA/RNA Viral Kit (Zymo Research, Irvine, CA, USA) and quality-checked with PCR targeting the mammalian mitochondrial cytochrome oxidase 1 (COX1; internal control) (34). Nucleic acid concentrations were measured with a Qubit 4 Fluorometer (Invitrogen) or spectrophotometer (MaestroGen). Up to 100 ng of DNA/RNA were used for each PCR reaction. The first screening for herpesviruses targeting a ~200 bp fragment of the herpesviral DNA polymerase gene (Dpol) was implemented from a previously published protocol (35). Positive and potential samples were then submitted to a second PCR protocol to amplify the ~500 bp fragment of the glycoprotein B gene (*GlyB*) (36). For all PCR assays, we used the Sapphire Amp Fast PCR Master Mix (Cat. No. RR350B, Takara Bio), with all protocols adapted. The PCR products were electrophoresed in 1% agarose gels. Fragments of the expected size were excised, and the gel was extracted using an E.Z.N.A.^®^ MicroElute^®^ Gel Extraction Kit (Omega Bio-tek) following the manufacturer's instructions. Products were then submitted for sequencing to the Plataformas Omicas UC, where they used an ABI PRISM 3500 xL DNA sequencer (Applied Biosystems^®^, USA). Sequences obtained were assembled with Geneious Prime v. 2022.2.2, and the primers were removed. The edited sequences were then submitted to GenBank, and BLASTN (megablast)/BLASTP (protein-protein BLAST) analyses were performed under a default setting to infer similarities with sequences available in GenBank.

### 2.3 Phylogenetic analysis

Phylogenetic analyses were performed with the amino acid sequences. Alignments were constructed for 59 herpesviruses and 44 sequences of Dpol and GlyB using MAFFT (37). An alphaherpesvirus was designated as the outgroup for the analysis. The phylogeny was inferred by Bayesian statistics with MrBayes 3.2.7a in the CIPRES science gateway (38). The trees were run for a maximum of 1,000,000 generations, with four chains, three hot chains, and one cold chain with the default heating parameter (temperature = 0.2), sampling every 100 generations, and the first 20% of the MCMC samples were discarded as burn-in. Figures were edited in FigTree v1.4.4 (39).

### 2.4 Data analysis

Positivity rate (prevalence) and 95% confidence intervals were estimated (when possible) using approximation and null hypothesis testing (positivity rate equals to 0) (40). Statistically significant differences were set at a *p*-value of < 0.05.

## 3 Results

Overall, 81 samples of blood were collected antemortem and 24 samples of spleen and three samples of lung were collected during the necropsy of pudus. Furthermore, five huemuls (two male and three female huemuls) were captured, and blood samples were collected as stated above. Most samples were obtained from individuals with no clinical signs of MCF at the time of sampling, except for 10 animals suspected to be clinically ill, including one animal with clinical signs consistent with MCF, such as fever (40.2°C temp), weakness, lethargy, and nasal mucous discharge.

All DNA extractions were positive for the *COX1* gene, with concentrations ranging from 31.5 to 56.2 ng/μL. Regarding herpesvirus detection, partial sequences were acquired from seven pudus and five huemuls, with a partial Dpol sequence identified in four pudus and five huemuls. Additionally, five pudus and four huemuls tested positive for the *GlyB* gene (Table 1).

**Table 1:** Samples of Chilean Cervids positive for herpesvirus.

**Animal**	**Sample**	**Genes**			**Results**		
		**Dpol**	**Cox1**	**GlyB**	**BLASTP results**	**GenBank accession number**	**GenBank accession number**
						**#Dpol**	**#GlyB**
20 Huemul	Blood	+	+	+	Pudu herpesvirus 1	OQ441122	OQ441125
21 Huemul	Blood	+	+	+	Pudu herpesvirus 1	OQ441122	OQ441125
22 Huemul	Blood	+	+	+	Pudu herpesvirus 1	OQ441122	OQ441125
23 Huemul	Blood	+	+	+^*^	Pudu herpesvirus 1	OQ441122	NA
24 Huemul	Blood	+	+	+	Pudu herpesvirus 1	OQ441122	OQ441125
61-2021 Pudu	Blood	+^*^	+	+	98.63% OvGHV-2	NA	OQ441127
91-2019 Pudu	Blood	+^*^	+	+	100% OvGHV-2	NA	OQ441126
84-2020 M Pudu	Blood	+	+	+	Pudu herpesvirus 1	OQ441122	OQ441124
68-2020 Pudu	Blood	+	+	+^*^	Pudu herpesvirus 1	OQ441122	NA
34-2022 Pudu	Blood	+	+	+	Pudu herpesvirus 1	OQ441122	OQ441124
106-21 Pudu	Blood	+^*^	+	+	Pudu herpesvirus 1	NA	OQ441124
CHS 142-2021 Pudu	Spleen	+	+	–	Pudu herpesvirus 1	OQ441122	NA

+ Positive; +^*^Band, but not sequence; – no band; NA, not applicable.

In the case of Dpol, a host species-specific consensus sequence of 175 bp was generated after primer removal for all positive pudus (GenBank accession OQ441122) and all positive huemuls (GenBank accession OQ441123). Pudu and huemul consensus and individual sequences were submitted for BlastN and BlastP analyses independently. In the case of pudu, BLASTN and BLASTP indicated that our sequences were 100% identical to pudu gammaherpesvirus-1 (LC667446.1/BDD79276.1). In the case of huemul, the consensus BLASTN indicated a 95.62% sequence identity to pudu gammhaerpesvirus-1 (LC667446.1). However, BLASTP analysis yielded a 100% sequence identity to pudu gammhaerpesvirus-1 (BDD79276.1), which indicated that both species were positive for the same pudu gammaherpesvirus-1. For the second gene, *GlyB*, we obtained a 467 bp length sequence from two pudus (GenBank accession OQ441124). Furthermore, we acquired sequences for three animals, all of which were exclusively identified as positive with GlyB. This contributes to the total of positive pudues by sequencing to seven (Table 2). One of them had a sequence that matches with pudu gammaherpesvirus-1. A second pudu with clinical signs consistent with MCF (GenBank accession OQ441127) had a 440 bp sequence with a 98.63% sequence identity with BLASTP to ovine gammaherpesvirus-2 (AAK28846.1), and the third animal (GenBank accession OQ441126) had a 484 bp sequence with a 100% sequence identity with BLASTP to ovine gammaherpesvirus-2. Finally, four of the five huemuls had a sequence for GlyB (GenBank accession OQ441125). In the GlyB of pudu gammhaerpesvirus-1, similar to Dpol, the sequences obtained had high intraspecies similarity with a 100% match at the amino acid level, among sequences of the same host. Consequently, the consensus sequences of the pudu samples and huemul samples were further evaluated. The BLASTP analysis of the pudu consensus sequence confirmed a 100% sequence identity with pudu gammaherpesvirus-1 (BDD79278.1). In the case of BLASTP, the analysis revealed a sequence identity of 99.24% to pudu gammaherpesvirus-1 (BDD79278.1). In this case, we identified an aminoacidic change from S to N at the 25th position of GlyB in our alignment. This result is consistent with the data obtained from the comparison of the pudu amino acid sequence obtained in the laboratory with 99.24% sequence identity and the same point of aminoacidic change from S to N. Pudu gammaherpesvirus-1 was detected in 9.90% (10/101; 95% CI = 5.11–17,87%) of the samples: all huemul specimens (100%; 5/5; 95% CI = 46.29–100%) and five pudus (5.20%; 5/96; 95% CI = 1.93–12.30%), with one pudu testing positive for ovine gammaherpesvirus-2 (1.04%; 1/96; 95% CI = 0.05–6.49%) and one pudu testing positive for a 98% compatible ovine gamma-herpesvirus-2 (1.04%; 1/96; 95% CI = 0.05–6.49%). Details related to the detection on the different sampled tissues are presented in Table 2.

**Table 2:** Herpesvirus-positive samples by species and tissue type.

**Species**	**Sample type**	**Sample size**	**Results (%; 95% CI)**
Pudu (*Pudu puda*)	Blood	81	6 (7.41; 3.05–16.01)
	Spleen	24	1 (4.17; 0.21–23.12)
	Lungs	3	0 (0; 0.00–69.00)
Huemul (*Hippocamelus bisulcus*)	Blood	5	5 (100; 46.29–100.00)

**Figure 1:**
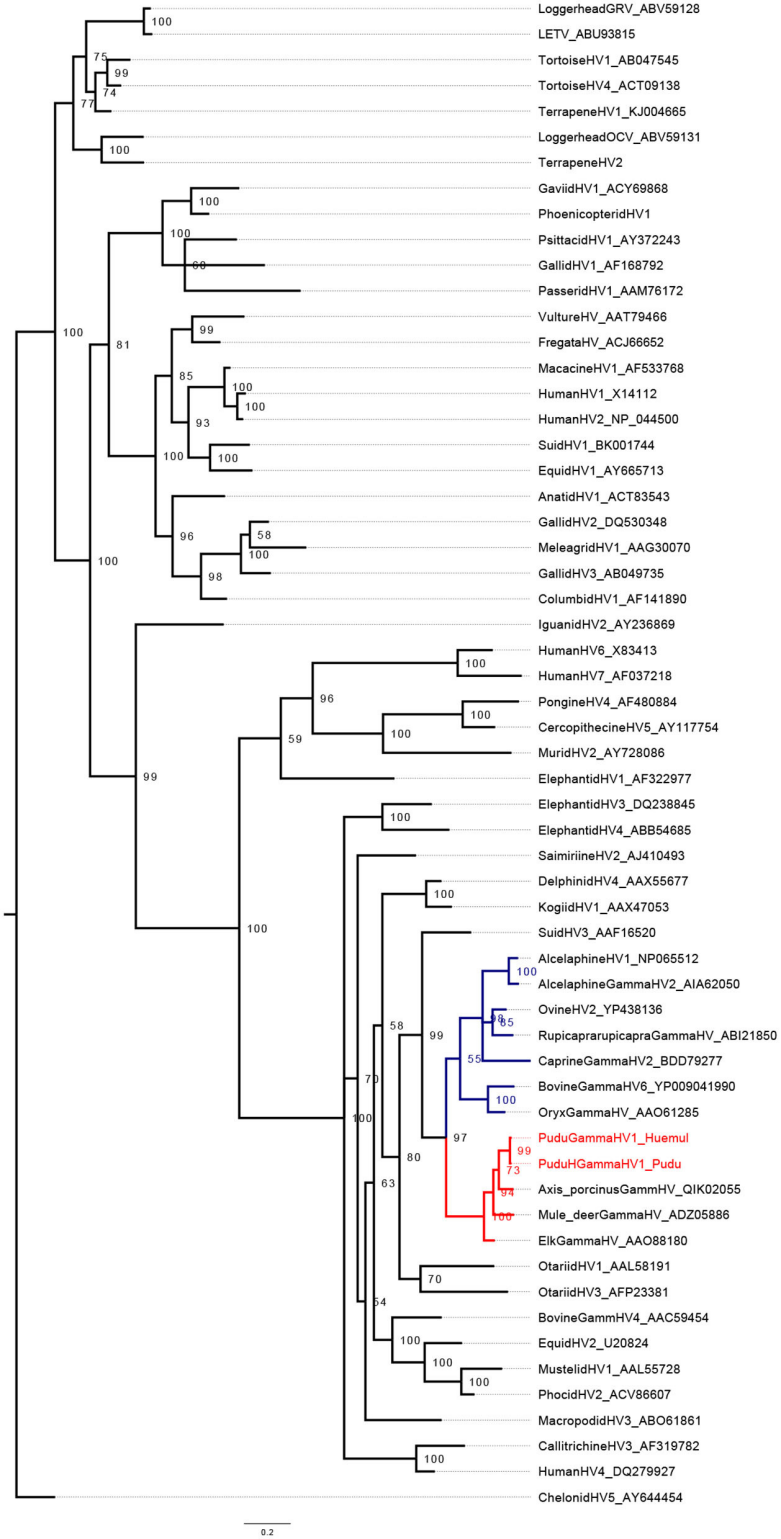
Midpoint-rooted Bayesian phylogenetic tree showing the relationship of pudu gammaherpesvirus-1 clustering in the genus *Rhadinovirus* (in red). The genus *Macavirus* is highlighted in blue. The analysis was carried out on amino acid (AA) sequences of partial herpes Dpol (176 AA characters including gaps).

**Figure 2:**
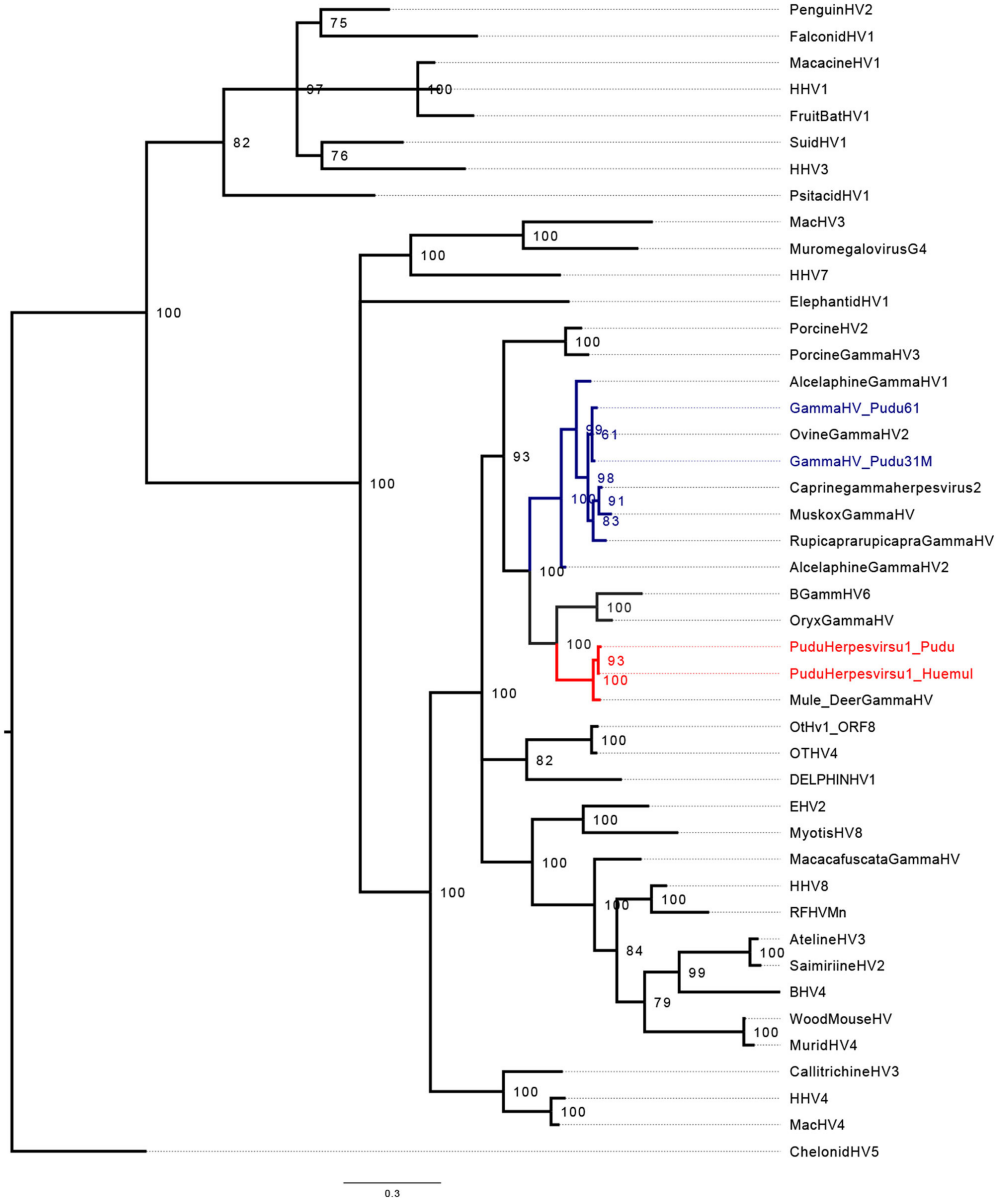
Midpoint-rooted Bayesian phylogenetic tree showing the relationship of pudu gammaherpesvirus-1 clustering in the genera *Rhadinovirus* (in red) and *Macavirus* (in blue). The analysis was carried out using the amino acid (AA) sequences for partial herpes GlyB (203 AA characters, including gaps and poorly aligned sequences).

Regarding phylogenetic reconstruction, pudu gammhaerpesvirus-1 clustered into a clade with *Rhadinovirus* (Figures 1, 2). The phylogenetic analysis for the *GlyB* gene indicates that the virus forms detected in pudu 91 2019 and pudu 61 2021 are clade within the *Macavirus* genus (Figure 2), and both are sister taxa of ovine gammaherpesvirus-2.

## 4 Discussion

This is the first systematic large-scale screening study conducted over a period of 5 years seeking herpesvirus DNA in any threatened free-ranging cervid species. This study describes the first report of ovine gammaherpesvirus-2 in Chilean ruminants. Our results confirmed that pudu gammaherpesvirus-1 is circulating in free-ranging Chilean pudus and huemuls (22). The occurrence of pudu gammaherpesvirus-1 in free-ranging huemuls in Torres del Paine National Park and in pudus of Chiloe, an island located ~1,100 km to the north, suggests that the detected virus circulates in Chilean wild deer despite the distance and geographic barriers between populations.

Previous molecular surveys of herpesvirus in cervid species showed variable levels of occurrence, ranging from 0.01% of ovine gammaherpesvirus-2 in sika deer (*Cervus nippon*) (19) in China to 4.7% of Elk gammaherpesvirus in Javan rusa (*Rusa timorensis*) in the USA (20). Both studies were conducted on animals under human care. In free-ranging cervids, the only comparative study was carried out among Eurasian tundra reindeer (*Rangifer tarandus tarandus*), moose (*Alces alces*), and red deer (*Cervus elaphus*), where herpesvirus was detected in 48.6% (17/35) of reindeers, while all moose and red deer were negative (2). The obtained sequences from this study were clearly different from other ruminant gammaherpesvirus sequences available from GenBank.

This is the first report of a pudu with clinical signs of MCF confirmed to be infected by a macavirus that is 98.63% similar to ovine gammaherpesvirus-2 for the *GlyB* gene. The pudu positive to with a to ovine gammaherpesvirus-2 was remitted dead to the rescue center as a consequence of an attack by dogs. However, no other clinical signs were observed on admission. Moreover, coinfections of different herpesvirus strains in the same individual were not observed. None of the screened samples from either pudu or huemul were positive for caprine gammaherpesvirus-2, suggesting that only deer in contact with goats, which are the natural reservoir of this virus, would be exposed to the contagion. Additional molecular surveys in goats of Patagonia will be necessary to rule out the caprine gammaherpesvirus-2 presence in the region.

Mortalities in captive or free-ranging artiodactyls associated with gammaherpesvirus are caused mainly by *Macavirus* and MCF (15, 16, 18–20, 41–51), suggesting that the current and future causes of mortality in both species of Chilean deer could be linked to infection by ovine gammaherpesvirus-2 or caprine gammaherpesvirus-2. The fact that fatal cases of infection by MCF have not been reported in Chilean cervids to date could be because of the inability to detect sick and dead animals in the field, calling for further efforts. A fluid communication between investigators and rescue centers that receive wild deer could be a solution to fill this gap. Notably, ovine gammaherpesvirus-2 usually causes diseases in domestic ruminants (8, 52, 53) and represents an emerging health threat to the herds of Chiloe Island and Chilean Patagonia.

The detection of pudu gammaherpesvirus-1 (100%) in five apparently healthy huemuls in Torres del Paine calls into question their origin and potential pathogenic role in Chilean cervids. Previously, the genus *Rhadinovirus* included all herpesviruses that cause MCF in cervids and other ungulates; in 2005, this clade was moved to the *Macavirus* genus by the International Committee on Taxonomy of Viruses. Currently, only one report of encephalitis induced by a newly discovered ruminant Rhadinovirus (RuRv) has been reported, in a free-ranging Formosan sambar deer (*Rusa unicolor swinhoei*) (54). It is notable that seven uteruses from hinds that were aborted were positive for cervid Rhadinovirus type-2 (CRhV-2) through PCR in a deer farm in New Zealand; however, the role of the virus in abortion needs clarification (55). Recent reports show novel Rhadinovirus sequences in cervid species with no evidence of disease (20, 56, 57); therefore, whether all Rhadinoviruses are pathogenic or not needs support. Finally, the pathogenicity and epidemiology of pudu gammaherpesvirus-1 in livestock is unknown. It is currently necessary to perform genetic screenings in sheep, goats, and cattle sharing the ecosystem with pudus from Patagonia to tackle this question.

## 5 Conclusion

A novel gammaherpesvirus, herein named pudu gammaherpesvirus-1, was detected in two species of cervids in Chilean Patagonia with no apparent health problems. Some gammaherpesviruses should be considered as a possible cause of illness in free-ranging pudus and domestic ruminants in Chilean Patagonia. The ovine gammaherpesvirus-2 detected in one rescued pudu with clinical signs of MCF could represent a risk for the health and conservation of the species due to its negative effects on wild populations as a direct (fatal infection) or indirect cause (weakness in the pudu that facilitates predation by feral dogs). Further studies, including serological and pathological screening, are needed to understand the diversity of herpesviruses in Chilean wild and domestic ruminants and their impact on animal health and conservation.

## Data availability statement

The datasets presented in this study can be found in online repositories. The names of the repository/repositories and accession number(s) can be found in the article/supplementary material.

## Ethics statement

Ethical approval was not required for the study involving animals in accordance with the local legislation and institutional requirements because this is not required for wild animal captures/management (Huemul) under the Chilean Legislation. The pudu samples proven from rehabilitation centers Frozen Bank.

## Author contributions

EH-H: Conceptualization, Data curation, Formal analysis, Funding acquisition, Investigation, Methodology, Project administration, Resources, Supervision, Writing – original draft, Writing – review & editing. JC: Data curation, Funding acquisition, Resources, Writing – review & editing. RL: Data curation, Funding acquisition, Investigation, Writing – review & editing. VV-W: Investigation, Resources, Writing – review & editing. FV: Data curation, Resources, Writing – review & editing. CV: Data curation, Resources, Writing – review & editing. CS: Funding acquisition, Investigation, Resources, Writing – original draft, Writing – review & editing. SC: Data curation, Funding acquisition, Resources, Writing – review & editing. AS: Resources, Writing – review & editing. AC: Investigation, Resources, Writing – review & editing. IB: Investigation, Resources, Writing – review & editing. RV: Data curation, Formal analysis, Investigation, Resources, Writing – review & editing. SM-L: Data curation, Resources, Writing – review & editing. PA: Data curation, Resources, Writing – review & editing. RL: Investigation, Resources, Writing – review & editing. JT-L: Investigation, Writing – review & editing. SV-C: Data curation, Investigation, Writing – original draft, Writing – review & editing. AG: Data curation, Investigation, Writing – original draft, Writing – review & editing. IP: Investigation, Methodology, Resources, Writing – review & editing. FS: Investigation, Resources, Writing – review & editing. DM-A: Investigation, Resources, Writing – review & editing. PV: Investigation, Resources, Writing – review & editing. RA-A: Formal analysis, Investigation, Methodology, Resources, Writing – original draft, Writing – review & editing. GC-H: Conceptualization, Data curation, Formal analysis, Funding acquisition, Investigation, Methodology, Project administration, Resources, Supervision, Validation, Writing – original draft, Writing – review & editing.
